# Supplementary material for: The association of hypertension among married Indian couples: a nationally representative cross-sectional study
Source: Sci Rep. 2024 May 6;14:10411. doi: 10.1038/s41598-024-61169-1 (PMC11074266; doi:10.1038/s41598-024-61169-1)
Supplement: Supplementary file 1 — Supplementary Information 1. [file 41598_2024_61169_MOESM1_ESM.docx]

**ONLINE- ONLY SUPPLEMENTARY MATERIAL**

[Supplementary Table 1. Characteristics of excluded sample 2](#_Toc164236528)

[Supplementary Table 2. Missingness in key covariates for analytic sample 3](#_Toc164236529)

[Supplementary Table 3. State-level marginal and co-occurrent prevalence of hypertension, n = 50,023 4](#_Toc164236530)

[Supplementary Table 4. Associations of hypertension status between couples for wives and husbands in India, adjusting for individual and shared household risk factors (Full model results) (n = 50,023) 6](#_Toc164236531)

[Supplementary Table 5. Association of spouse’s hypertension with own hypertension status adjusting for spouse’s characteristics, n = 50,023 7](#_Toc164236532)

[Supplementary Table 6. Association of spouse’s blood pressure with own blood pressure, n = 50,023 couples 8](#_Toc164236533)

[Supplementary Table 7. Association of spouse’s hypertension status with own hypertension status in overall sample before exclusion by missing biomarkers, n = 54,356 9](#_Toc164236534)

[Supplementary Note 1. Spousal concordance and joint burden of disease 10](#_Toc164236535)

[Supplementary Figure 1. Flowchart of analytic sample 12](#_Toc164236536)

[Supplementary Figure 2. Framework for analysis of spousal concordance 13](#_Toc164236537)

[Supplementary Figure 3. State-level prevalence of hypertension burden among spousal pairs 14](#_Toc164236538)

[Supplementary Figure 4. Total and undiagnosed hypertension in married Indian adults by spouse’s hypertension status, n = 50,023 15](#_Toc164236539)

# Supplementary Table 1. Characteristics of excluded sample

|  | **Excluded sample (n = 4,333)** | |
| --- | --- | --- |
|  | **Husbands** | **Wives** |
| **Individual characteristics** |  |  |
| Age in years | 39.2 (38.9, 39.6) | 34.4 (34, 34.7) |
| Years of schooling | 8.6 (8.4, 8.9) | 7.3 (7, 7.5) |
| Consumes alcohol (%) | 28.1 (26.1, 30.2) | 1.1 (0.7, 1.4) |
| Tobacco use (%) | 47.6 (45.2, 49.9) | 4.8 (3.9, 5.6) |
| Body mass index (kg/m^2^) | 23.9 (23.6, 24.1) | 23.8 (23.5, 24.0) |
| ***Weight status*** |  |  |
| Underweight (< 18.5kg/m^2^) | 2.9 (2.3, 3.6) | 8.6 (7.5, 9.7) |
| Normal (18.5 – 24.9kg/m^2^) | 21.3 (19.6, 23) | 37.2 (35.3, 39.2) |
| Overweight (25-29.9kg/m^2^) | 11.1 (9.9, 12.4) | 18.5 (16.9, 20.1) |
| Obese (≥ 30 kg/m^2^) | 2.5 (1.9, 3.1) | 7.1 (6.1, 8.1) |
| ***Attained schooling*** |  |  |
| No education | 14.2 (12.9, 15.6) | 26.4 (24.7, 28.2) |
| Primary | 13.3 (11.7, 14.9) | 12.6 (11.2, 14.1) |
| Secondary | 51.4 (49.2, 53.6) | 44.6 (42.3, 46.9) |
| Higher | 21 (19, 23.1) | 16.3 (14.2, 18.4) |
| **Household characteristics** |  |  |
| Number of children | 5.5 (5.4, 5.6) | *To be merged* |
| Household members | 2.3 (2.3, 2.4) |  |
| ***Household wealth quintile at national level (%)*** |  |  |
| Q1 | 14.7 (13.3, 16.1) | *To be merged* |
| Q2 | 15.8 (14.3, 17.3) |  |
| Q3 | 18.5 (16.8, 20.1) |  |
| Q4 | 22.2 (20.4, 24) |  |
| Q5 | 28.8 (26.5, 31.2) |  |
| ***Rural (%)*** | 56.7 (53.7, 59.7) |  |
| **Chronic disease outcomes** |  |  |
| Diabetes (%) | - | - |
| *Self-reported Diabetes (%)* | - | - |
| *Treated Diabetes (%)* | - | - |
| ***Joint prevalence of Diabetes (%)*** |  |  |
| Hypertension (%) | - | - |
| *Self-reported Hypertension (%)* | - | - |
| *Treated Hypertension (%)* | - | - |
| ***Joint prevalence Hypertension (%)*** |  |  |

# Supplementary Table 2. Missingness in key covariates for analytic sample

|  | **Analytic sample (n = 50,023)** | |
| --- | --- | --- |
|  | **Husbands** | **Wives** |
| **Individual characteristics** |  |  |
| Age in years | 0 (0%) | 0 (0%) |
| Years of schooling | 0 (0%) | 0 (0%) |
| Consumes alcohol (%) | 0 (0%) | 0 (0%) |
| Tobacco use (%) | 0 (0%) | 0 (0%) |
| Body mass index (kg/m^2^) | 155 (0.3%) | 86 (0.2%) |
| ***Attained schooling*** | 0 (0%) | 0 (0%) |
| **Household characteristics** |  |  |
| Number of children | 0 (0%) |  |
| Household members | 0 (0%) |  |
| ***Household wealth quintile at national level (%)*** | 0% |  |
| ***Rural (%)*** | 0% |  |
| ***Caste endogamy (%)*** | 0 (0%) |  |
| **Chronic disease outcomes** |  |  |
| Random Blood Glucose (mg/dL) |  |  |
| Systolic Blood Pressure (mm Hg) | 9 (0%) | 1 (0%) |
| Diastolic Blood Pressure (mm Hg) | 9 (0%) | 1 (0%) |
| Diabetes (%) |  |  |
| *Self-reported Diabetes (%)* | 0 (0%) | 0 (0%) |
| *Co-occurring Diabetes (%)* |  |  |
| Hypertension (%) |  |  |
| *Self-reported Hypertension (%)* | 0 (0%) | 0 (0%) |
| *Co-occurring Hypertension (%)* |  |  |

# Supplementary Table 3. State-level marginal and co-occurrent prevalence of hypertension, n = 50,023

|  | **Husbands** | **Wives** | **Co-occurent** |
| --- | --- | --- | --- |
| Andaman & Nicobar Islands | 38.8  (30.3, 47.3) | 17.6  (11.5, 23.7) | 9.3  (4.3, 14.4) |
| Andhra Pradesh | 30.3  (26.9, 33.7) | 19.5  (16.4, 22.6) | 8.5  (6.6, 10.5) |
| Arunachal Pradesh | 41.7  (37.9, 45.5) | 30.2  (26.9, 33.5) | 16.3  (13.5, 19.1) |
| Assam | 26.5  (24.4, 28.6) | 19  (17.1, 20.8) | 7  (5.9, 8.2) |
| Bihar | 26.5  (23.7, 29.4) | 21.7  (19.1, 24.3) | 9.6  (7.6, 11.6) |
| Chandigarh | 38.8  (24.4, 53.2) | 28.2  (14.2, 42.2) | 9.5  (1.4, 17.5) |
| Chhattisgarh | 33.2  (30.6, 35.8) | 22.3  (20.1, 24.5) | 9.3  (7.5, 11.1) |
| Dadra & Nagar Haveli And Daman & Diu | 15.7  (8.8, 22.6) | 14.2  (9.2, 19.2) | 1.2  (-0.1, 2.5) |
| Goa | 32.4  (23.4, 41.5) | 15.9  (9.7, 22.2) | 6.7  (2.3, 11.2) |
| Gujarat | 20.9  (18.6, 23.2) | 15.7  (13.9, 17.5) | 5.2  (4.1, 6.2) |
| Haryana | 31.8  (28.7, 34.8) | 27.7  (24.4, 30.9) | 11  (8.8, 13.1) |
| Himachal Pradesh | 29.8  (25.1, 34.4) | 20.8  (16.9, 24.7) | 9.6  (6.8, 12.5) |
| Jammu & Kashmir | 24.2  (20.8, 27.6) | 23.1  (19.6, 26.6) | 8.7  (6.5, 11) |
| Jharkhand | 29  (26.3, 31.6) | 19.8  (16.8, 22.9) | 9  (6.8, 11.2) |
| Karnataka | 34.6  (31.5, 37.8) | 17.9  (15.9, 19.9) | 7.6  (6, 9.2) |
| Kerala | 32.6  (28.6, 36.6) | 18.2  (14.7, 21.6) | 7.3  (5.2, 9.4) |
| Ladakh | 26.5  (15.4, 37.6) | 25.4  (14.2, 36.7) | 12.4  (1.1, 23.6) |
| Lakshadweep | 30.5  (16.8, 44.2) | 23  (12.5, 33.5) | 9.3  (0.6, 18) |
| Madhya Pradesh | 27.2  (25.2, 29.2) | 20  (18.3, 21.6) | 7.1  (6.1, 8.1) |
| Maharashtra | 27.6  (24.9, 30.3) | 19.2  (16.7, 21.7) | 7.1  (5.2, 8.9) |
| Manipur | 44.7  (38.9, 50.5) | 24.9  (21.2, 28.7) | 13.1  (9.2, 17.1) |
| Meghalaya | 28.9  (22.3, 35.6) | 21  (15.7, 26.3) | 8.6  (4.8, 12.5) |
| Mizoram | 27.5  (20.8, 34.3) | 18.4  (13.6, 23.2) | 5.1  (2.5, 7.7) |
| Nagaland | 35.2  (30, 40.4) | 27.8  (22.7, 33) | 10.9  (7.6, 14.2) |
| Nct Of Delhi | 46.7  (41.8, 51.6) | 23.8  (20.1, 27.5) | 12.8  (10.2, 15.4) |
| Odisha | 30.9  (28.3, 33.4) | 23  (20.7, 25.3) | 8.8  (7.2, 10.3) |
| Puducherry | 32  (21.4, 42.7) | 11.6  (4.9, 18.4) | 3.2  (0.6, 5.9) |
| Punjab | 49.5  (46, 52.9) | 38  (34.4, 41.7) | 21.5  (18.4, 24.7) |
| Rajasthan | 23.1  (21, 25.1) | 16.8  (15, 18.5) | 6.7  (5.5, 7.9) |
| Sikkim | 53.8  (41.9, 65.8) | 38.1  (28.8, 47.3) | 24.2  (15.5, 32.9) |
| Tamil Nadu | 36.3  (33.5, 39.1) | 20.5  (17.7, 23.4) | 10.2  (8, 12.5) |
| Telangana | 35.9  (32.9, 39) | 19.3  (17.1, 21.4) | 8.9  (7.3, 10.4) |
| Tripura | 28.9  (23.7, 34) | 22.5  (18.9, 26.2) | 9  (5.9, 12.1) |
| Uttar Pradesh | 29.1  (27.5, 30.7) | 24  (22.6, 25.5) | 9.4  (8.4, 10.5) |
| Uttarakhand | 41.7  (37, 46.3) | 25.9  (21.5, 30.3) | 12.6  (8.6, 16.5) |
| West Bengal | 22.8  (20.2, 25.3) | 18.4  (16.1, 20.8) | 6  (4.4, 7.6) |
| Andaman & Nicobar Islands | 38.8  (30.3, 47.3) | 17.6  (11.5, 23.7) | 9.3  (4.3, 14.4) |

Survey weighted prevalence estimates and 95% confidence intervals

# Supplementary Table 4. Associations of hypertension status between couples for wives and husbands in India, adjusting for individual and shared household risk factors (Full model results) (n = 50,023)

|  | **Hypertension** | |
| --- | --- | --- |
| ***Prevalence Ratio (95% CI)*** | **Wives** | **Husbands** |
| Spouse’s confirmed disease status | 1.37  (1.30, 1.44) | 1.32  (1.26, 1.38) |
| ***Own characteristics*** |  |  |
| Age in years | 1.05  (1.04, 1.05) | 1.03  (1.03, 1.04) |
| Level of education (ref: No education) |  |  |
| Primary | 1.03  (0.96, 1.11) | 1.06  (0.99, 1.14) |
| Secondary | 0.99  (0.93, 1.06) | 1.08  (1.02, 1.15) |
| Higher | 0.87  (0.77, 0.98) | 1.11  (1.03, 1.21) |
| Consumes alcohol (%) | 1.20  (1.04, 1.40) | 1.21  (1.16, 1.27) |
| Tobacco use (%) | 1.14  (1.04, 1.25) | 0.99  (0.95, 1.04) |
| Body mass index (kg/m^2^) | 1.05  (1.05, 1.06) | 1.04  (1.03, 1.04) |
| ***Household characteristics*** |  |  |
| Number of children (count) | 0.97  (0.95, 0.99) | 1.00  (0.98, 1.01) |
| Household members (count) | 1.00  (0.99, 1.01) | 0.99  (0.99, 1.00) |
| Rural residence | 1.04  (0.98, 1.11) | 0.92  (0.87, 0.97) |
| Consanguineous marriage | 1.12  (1.04, 1.21) | 1.00  (0.94, 1.06) |
| National Wealth Quintile (ref: Q1) |  |  |
| Q2 | 1.13  (1.04, 1.22) | 1.02  (0.96, 1.08) |
| Q3 | 1.04  (0.97, 1.13) | 1.01  (0.95, 1.08) |
| Q4 | 1.04  (0.95, 1.12) | 1.06  (0.99, 1.13) |
| Q5 | 1.05  (0.96, 1.14) | 1.06  (0.99, 1.13) |

*Note: the spousal associations from this model are reported in Table 2 of the main manuscript

# Supplementary Table 5. Association of spouse’s hypertension with own hypertension status adjusting for spouse’s characteristics, n = 50,023

|  | **Hypertension** | |
| --- | --- | --- |
| ***Prevalence Ratio (95% CI)*** | **Wife** | **Husband** |
| Spouse’s confirmed disease status | 1.38 (1.31, 1.46) | 1.31 (1.25, 1.37) |
| ***Personal characteristics*** |  |  |
| Age (years) | 1.05 (1.04, 1.05) | 1.03 (1.03, 1.04) |
| Level of education (ref: None) |  |  |
| Primary | 1.03 (0.95, 1.11) | 1.05 (0.98, 1.13) |
| Secondary | 1.00 (0.94, 1.08) | 1.08 (1.02, 1.15) |
| Higher | 0.91 (0.79, 1.04) | 1.14 (1.04, 1.24) |
| Alcohol use | 1.22 (1.05, 1.41) | 1.21 (1.16, 1.27) |
| Tobacco use | 1.14 (1.04, 1.25) | 0.99 (0.94, 1.04) |
| BMI (kg/m^2^) | 1.05 (1.05, 1.06) | 1.04 (1.03, 1.04) |
| ***Household characteristics*** |  |  |
| Number of children (count) | 0.97 (0.95, 0.99) | 1.00 (0.98, 1.01) |
| Household members (count) | 1.00 (0.99, 1.01) | 0.99 (0.99, 1.00) |
| Rural residence | 1.03 (0.97, 1.10) | 0.91 (0.87, 0.97) |
| Consanguineous marriage | 1.12 (1.03, 1.20) | 1.00 (0.94, 1.06) |
| Regional Wealth Quintile (ref: Q1) |  |  |
| Q2 | 1.13 (1.04, 1.22) | 1.02 (0.96, 1.08) |
| Q3 | 1.05 (0.97, 1.14) | 1.01 (0.95, 1.08) |
| Q4 | 1.05 (0.97, 1.14) | 1.06 (1.00, 1.13) |
| Q5 | 1.07 (0.98, 1.18) | 1.06 (0.99, 1.14) |
| ***Spouse’s characteristics*** |  |  |
| Level of education (ref: None) |  |  |
| Primary | 1.05 (0.97, 1.14) | 1.04 (0.98, 1.11) |
| Secondary | 1.01 (0.94, 1.09) | 1.00 (0.95, 1.06) |
| Higher | 0.96 (0.86, 1.08) | 0.95 (0.86, 1.04) |
| Alcohol use | 0.98 (0.93, 1.04) | 1.00 (0.89, 1.13) |
| Tobacco use | 1.04 (0.99, 1.09) | 1.06 (0.98, 1.16) |
| BMI (kg/m^2^) | 0.99 (0.99, 1.00) | 1.00 (1.00, 1.01) |

Age (years) for spouse was not included due to multicollinearity

*Note: the spousal associations from this model are reported in Table 2 of the main manuscript

# Supplementary Table 6. Association of spouse’s blood pressure with own blood pressure, n = 50,023 couples

|  | **Systolic Blood Pressure (mm Hg)** | |
| --- | --- | --- |
| ***Coefficient (95% CI)*** | **Wives** | **Husbands** |
| Spouse’s biomarker (per 1 unit) | 0.12  (0.11, 0.13) | 0.13  (0.11, 0.14) |
| ***Personal characteristics*** |  |  |
| Age in years | 0.44  (0.42, 0.46) | 0.20  (0.18, 0.22) |
| Level of education (ref: No education) |  |  |
| Primary | -0.81  (-1.33, -0.29) | 0.20  (-0.38, 0.78) |
| Secondary | -1.12  (-1.56, -0.68) | 0.37  (-0.12, 0.86) |
| Higher | -2.02  (-2.70, -1.35) | 0.03  (-0.61, 0.67) |
| Consumes alcohol (%) | 1.99  (0.74, 3.23) | 1.72  (1.34, 2.10) |
| Tobacco use (%) | -0.68  (-1.39, 0.04) | -0.56  (-0.94, -0.18) |
| Body mass index (kg/m^2^) | 0.46  (0.42, 0.50) | 0.57  (0.52, 0.62) |
| ***Household characteristics*** |  |  |
| Number of children (count) | -0.63  (-0.78, -0.49) | 0.02  (-0.12, 0.16) |
| Household members (count) | 0.01  (-0.05, 0.06) | -0.03  (-0.08, 0.02) |
| Rural residence | 0.45  (-0.01, 0.92) | -0.74  (-1.19, -0.29) |
| Regional Wealth Quintile (ref: Q1) |  |  |
| Q2 | 0.19  (-0.32, 0.71) | -0.06  (-0.56, 0.45) |
| Q3 | 0.20  (-0.31, 0.71) | 0.37  (-0.13, 0.87) |
| Q4 | 0.10  (-0.45, 0.65) | 0.34  (-0.21, 0.90) |
| Q5 | 0.15  (-0.46, 0.76) | 0.56  (-0.02, 1.14) |

# Supplementary Table 7. Association of spouse’s hypertension status with own hypertension status in overall sample before exclusion by missing biomarkers, n = 54,356

|  | **Hypertension** | |
| --- | --- | --- |
| ***Prevalence Ratio (95% CI)*** | **Wives** | **Husbands** |
| Spouse’s confirmed disease status | 1.37  (1.30, 1.45) | 1.31  (1.26, 1.37) |
| ***Personal characteristics*** |  |  |
| Age in years | 1.05  (1.04, 1.05) | 1.04  (1.03, 1.04) |
| Level of education (ref: No education) |  |  |
| Primary | 1.04  (0.97, 1.12) | 1.06  (0.99, 1.13) |
| Secondary | 0.99  (0.93, 1.06) | 1.09  (1.03, 1.15) |
| Higher | 0.84  (0.75, 0.94) | 1.12  (1.04, 1.21) |
| Consumes alcohol (%) |  |  |
| Tobacco use (%) | 1.11  (1.02, 1.21) | 0.98  (0.94, 1.03) |
| Body mass index (kg/m^2^) | 1.03  (1.02, 1.03) | 1.01  (1.01, 1.02) |
| ***Household characteristics*** |  |  |
| Number of children (count) | 0.97  (0.95, 0.99) | 1.00  (0.99, 1.02) |
| Household members (count) | 1.00  (0.99, 1.01) | 0.99  (0.99, 1.00) |
| Rural residence | 1.04  (0.98, 1.12) | 0.96  (0.91, 1.01) |
| National Wealth Quintile (ref: Q1) |  |  |
| Q2 | 1.02  (0.95, 1.10) | 1.07  (1.01, 1.14) |
| Q3 | 1.09  (1.01, 1.18) | 1.17  (1.10, 1.24) |
| Q4 | 1.13  (1.04, 1.24) | 1.20  (1.12, 1.29) |
| Q5 | 1.09  (0.99, 1.21) | 1.20  (1.11, 1.30) |

Analysis was carried out for all couples of legal age with and without the outcome variable after imputing the outcome variable and not deleting after imputation. All associations (95% confidence intervals) were estimated from survey-weighted Poisson regression (with robust standard errors) of 10 multiply-imputed datasets. Associations were adjusted for state of residence.

# Supplementary Note 1. Spousal concordance and joint burden of disease

|  | **Husband’s Outcome = Yes** | **Husband’s Outcome = No** | ***Marginal*** |
| --- | --- | --- | --- |
| **Wife’s Outcome = Yes** | Pr[W=1, H = 1] | Pr[W=1, H = 0] | Pr[W = 1] |
| **Wife’s Outcome = No** | Pr[W=0, H = 1] | Pr[W=0, H = 0] | Pr[W = 0] |
| ***Marginal*** | Pr[H = 1] | Pr[H = 0] |  |

**Method 1: Used in the current manuscript**

From the regression model, we are estimating the magnitude of couple concordance as the prevalence ratio (PR) of disease based on the spouse’s disease status based on a Poisson regression, i.e.

PR = Pr[W=1|H=1] ÷ Pr[W=1|H=0]

= Pr[W = 1,H = 1]/Pr[H = 1] ÷ Pr[W = 1, H = 0]/Pr[H=0]

We estimate the 95% robust confidence intervals for the prevalence ratios. As the marginal prevalence increases across both margins, a greater difference between observed and expected (product of margins) joint prevalence is required to achieve the same magnitude of spousal association of disease. We consider this to be a statistical issue related to differences in marginal prevalence and not related to stronger or weaker spousal concordance since spouses are still more likely than independent pairs to be concordant in disease status.

**Method 2**

Tests of spousal concordance are under a null hypothesis of Pr[W=1] = Pr[H = 1] or Pr[W=0, H =1] = Pr[W=1, H =0] based on a contingency table such as the one above using McNemar’s Chi-squared test for paired samples. The cell proportions are proportions of husband-wife pairs with the observed joint burden. The test statistic is calculated based on the numbers of discordant pairs in the contingency matrix.

McNemar’s test statistic:

χ^2^_(df=1)_ = (N_W=0, H = 1_ – N_W=1, H = 0_)^2^ ÷ (N_W=0, H = 1_ + N_W=1, H = 0_)

**Method 3**

If we were to perform Pearson’s chi-squared test for independence, the test may be a slightly less powerful test for equality of proportions, but these differences may not matter for large sample sizes:

χ^2^_(df=1)_ = $\sum\frac{{(N_{W=1, H=1}-N.\Pr\left[ W=1 \right].Pr[H=1])}^{2}}{N.\Pr\left[ W=1 \right].Pr[H=1]}$

# Supplementary Figure 1. Flowchart of analytic sample


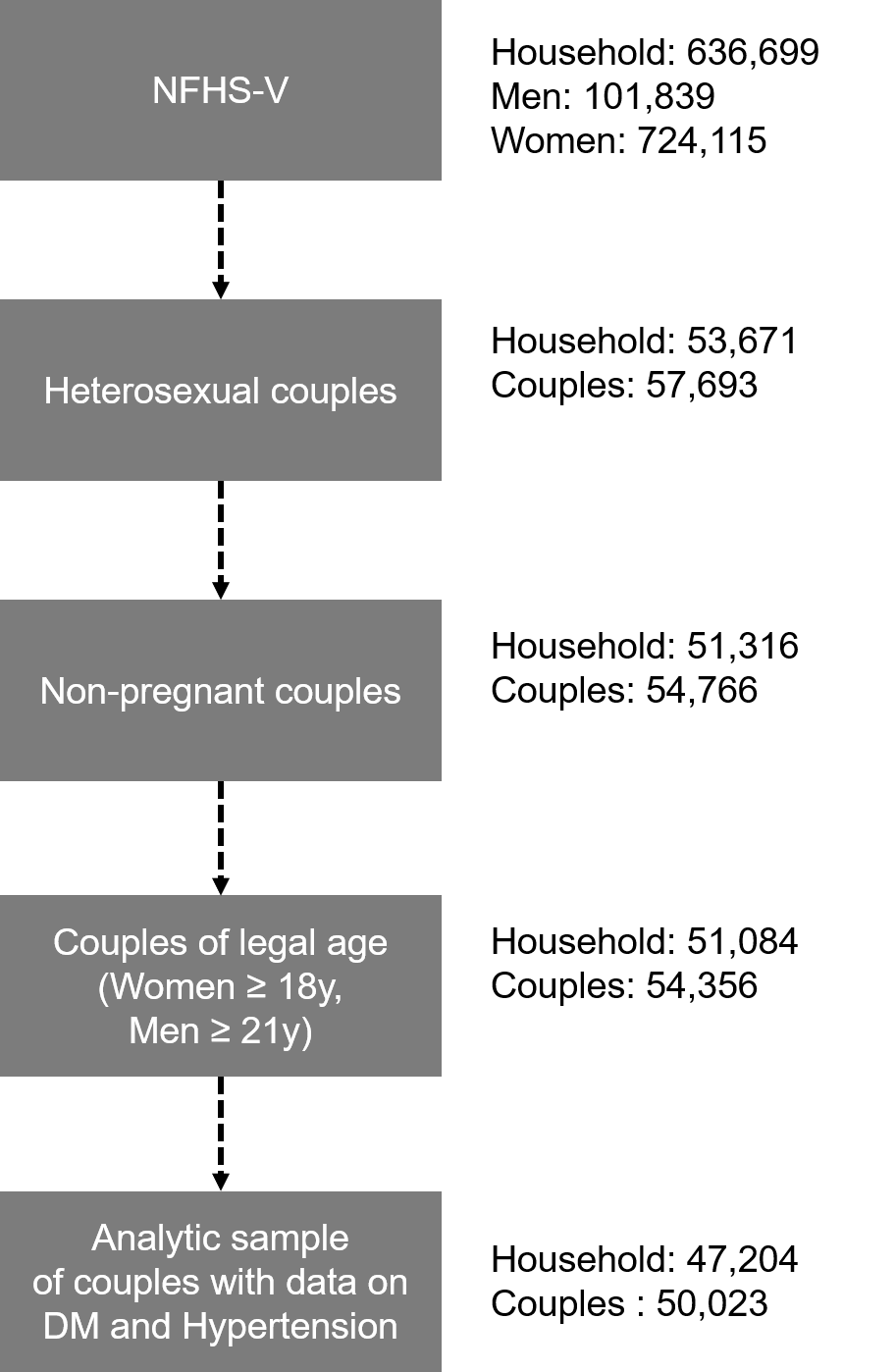


# Supplementary Figure 2. Framework for analysis of spousal concordance


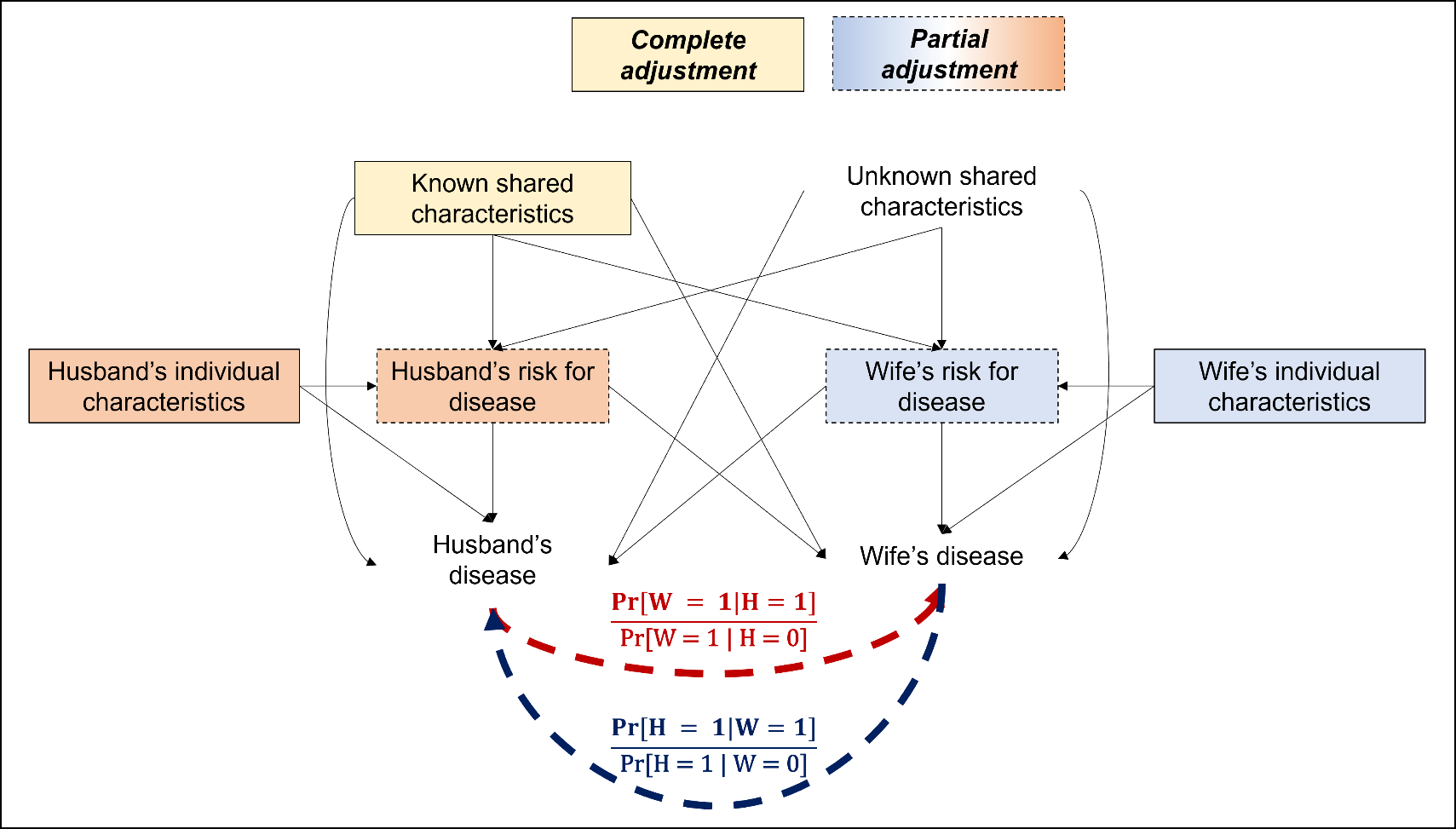


Unknown shared characteristics include shared genetics, shared environment before marriage (e.g. built environment, diet and physical activity) and shared environment after marriage that are confounders for the observed spousal concordance. This is in addition to other pathways such as marital satisfaction and physiological responses from relationship quality that could be from spouse’s disease. The association of husband’s disease status with wife’s disease status is shown in Red, and requires adjustment for wife’s individual characteristics and known shared characteristics. The association of wife’s disease status with husband’s disease status is shown in blue and requires adjustment for husband’s individual characteristics and known shared characteristics.

# Supplementary Figure 3. State-level prevalence of hypertension burden among spousal pairs


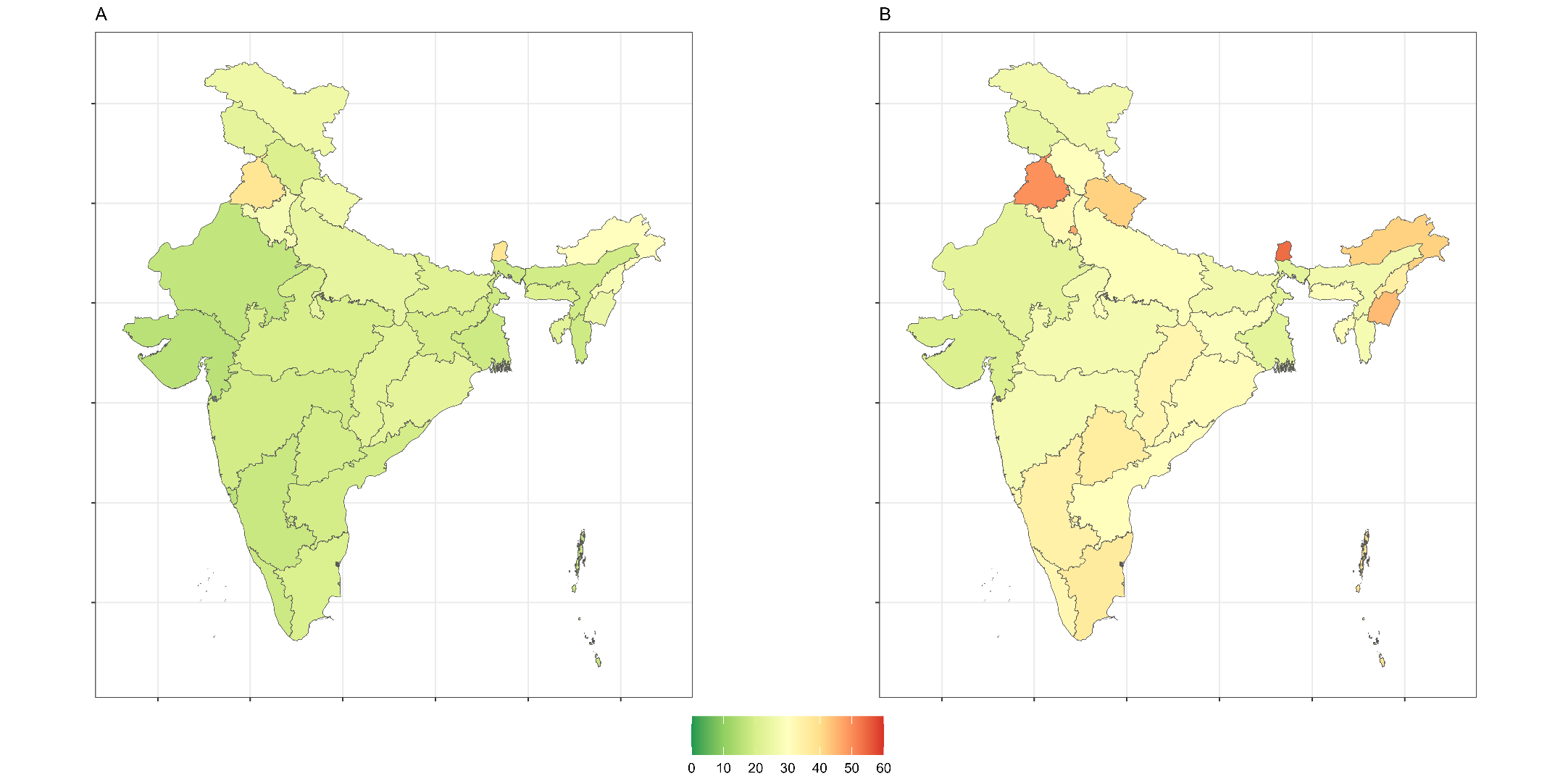


A: Wives hypertension, B: Husband’s hypertension. The above figure was generated using the R package ggplot2 version 3.4.2.

# Supplementary Figure 4. Total and undiagnosed hypertension in married Indian adults by spouse’s hypertension status, n = 50,023


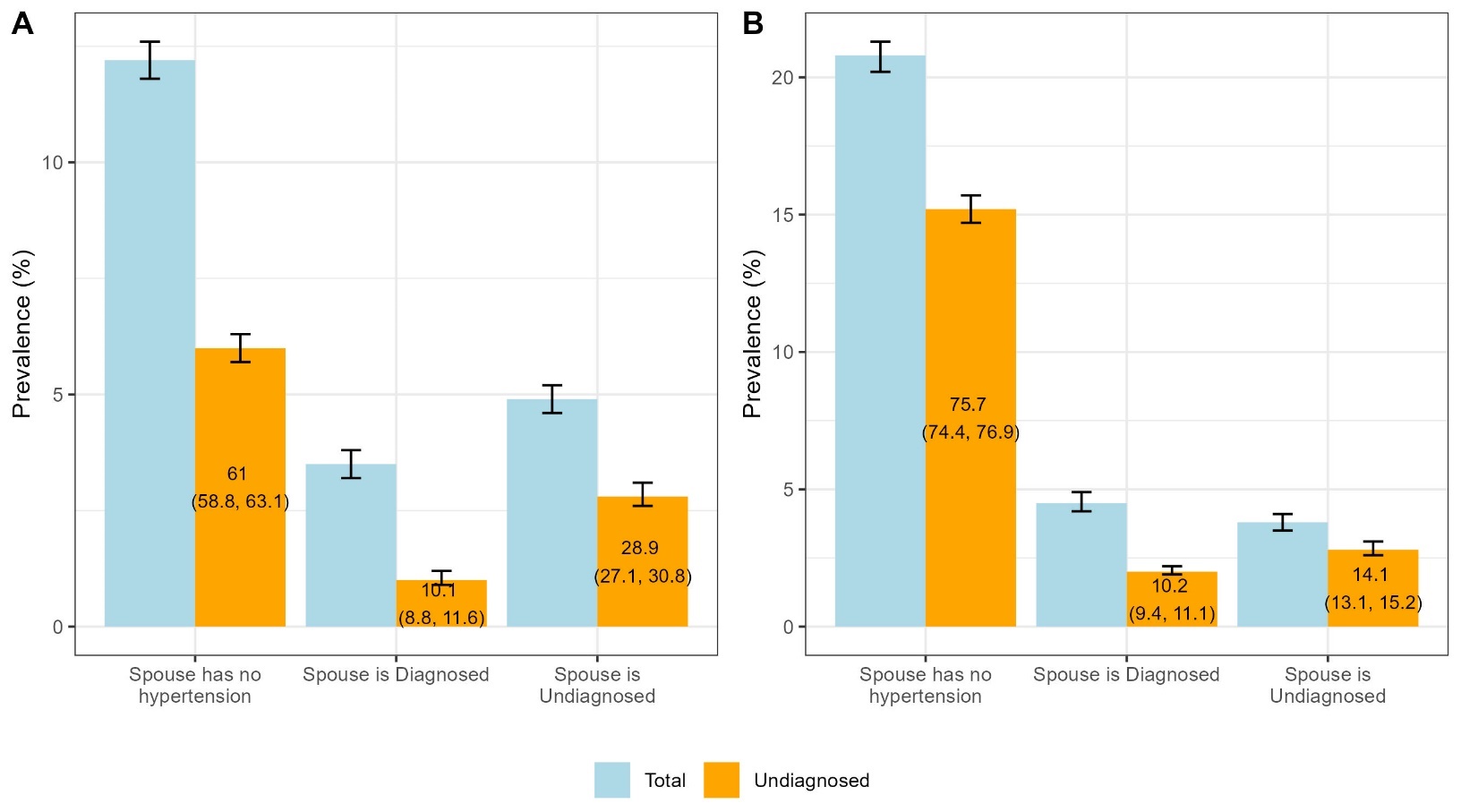


All estimates are proportions and 95% confidence intervals. Estimates in columns of undiagnosed diabetes are proportions attributed to hypertension status of their spouse, (**A**) among married women by husband’s hypertension diagnosis status and (**B**) Among married men by wife’s hypertension diagnosis status

**Supplementary Figure 5. Concordance of hypertension status in married Indian couples, n = 50,013**


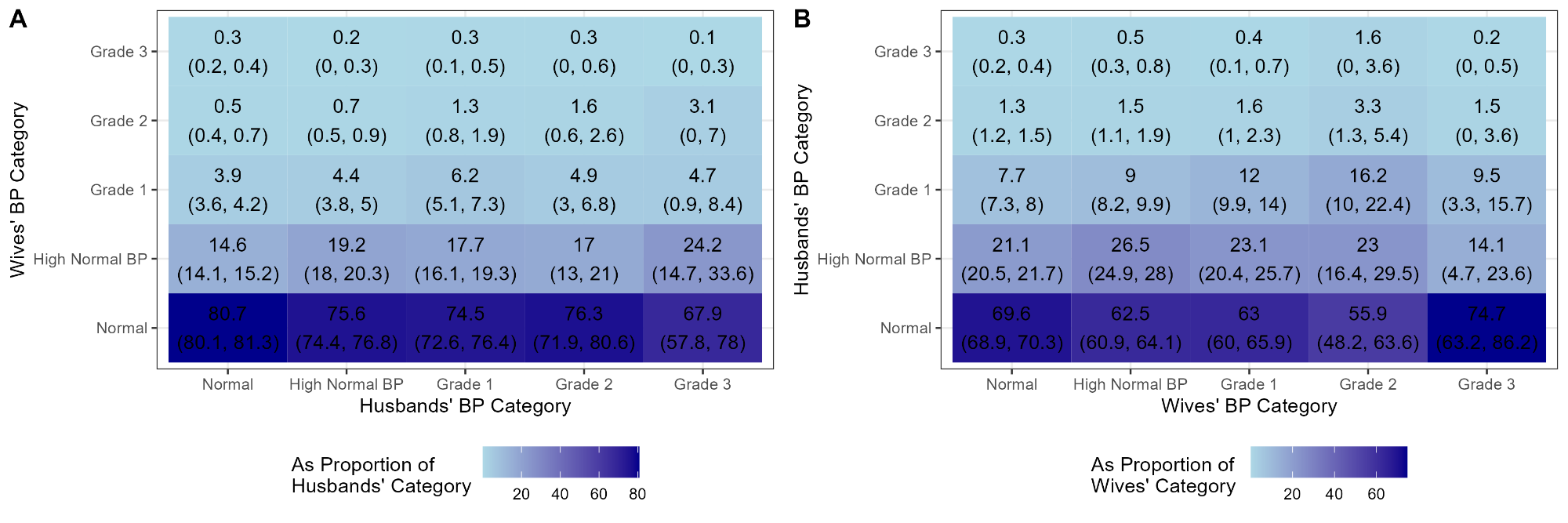


Panel A and Panel B correspond to conditional probably for each category relative to Husbands’ and Wives’ status. The conditional probability of both partners in a couple belonging to the same category ‘k’ (*diagonal*) is higher than the conditional probability of an individual belonging to that category ‘k’ when the spouse belongs to ‘Normal’ category (*Column 1*) for all categories except Grade 3 hypertension, i.e. Pr[W_k_ = 1|H_k_ = 1]/Pr[W_k_=1|H_1_=1] > 1. Observations (N=10) with missing values of either SBP or DBP for husband or wife were omitted.
